# Single nucleotide polymorphisms of the tenomodulin gene (*TNMD*) in age-related macular degeneration

**Published:** 2009-04-15

**Authors:** Anna-Maija Tolppanen, Tanja Nevalainen, Marjukka Kolehmainen, Sanna Seitsonen, Ilkka Immonen, Matti Uusitupa, Kai Kaarniranta, Leena Pulkkinen

**Affiliations:** 1 Department of Clinical Nutrition and Food and Health Research Centre, School of Public Health and Clinical Nutrition, University of Kuopio, Kuopio, Finland; 2 Department of Ophthalmology, Kuopio University Hospital, Kuopio, Finland; 3 Department of Ophthalmology, Helsinki University Hospital, Helsinki, Finland

## Abstract

**Purpose:**

Tenomodulin (*TNMD)* is located in the X-chromosome encoding a putative angiogenesis inhibitor which is expressed in retina. Associations of single nucleotide polymorphisms of *TNMD* with the prevalence of age-related macular degeneration (AMD) were examined.

**Methods:**

Six markers covering 75% of the common sequence variation in the coding region of *TNMD* and 10 kb up- and downstream were genotyped in a sample consisting of 89 men and 175 women with exudative AMD, 18 men and 25 women with atrophic AMD, and 55 men and 113 women without AMD. All participants were over 65 years old and did not have diabetes mellitus. Due to the chromosomal locus, the association of genotypes with AMD was assessed genderwise.

**Results:**

Three markers, rs1155974, rs2073163, and rs7890586, were associated with a risk of AMD in women. In comparison to women with other genotypes, the women who were homozygous for the minor allele (genotypes rs1155974-TT or rs2073163-CC) had 2.6 fold (p=0.021) or 1.9 fold (p=0.067) risk for having AMD, respectively. These differences were due to the unequal prevalence of exudative AMD. In comparison to women who were homozygous for the major alleles, the women with rs1155974-TT genotype had a 2.8 fold risk (p=0.021 in additive model; p=0.022 in recessive model) for exudative AMD, and the women with rs2073163-CC genotype had a 1.8 fold risk (p=0.09 in additive model; p=0.038 in recessive model). Furthermore, women carrying the rare rs7890586-AA genotype had a significantly smaller risk for having AMD than women with the other genotypes (odds ratio 0.083; p=0.001 in recessive model), but due to the low frequency of this genotype, this finding must be interpreted cautiously. The false discovery rate was <10% for all of the aforementioned results.

**Conclusions:**

On the basis of the putative antiangiogenic role of TNMD and the present genetic associations of *TNMD* with AMD in women, we suggest that *TNMD* could be a novel candidate gene for AMD. These results should be confirmed in further studies.

## Introduction

Age-related macular degeneration (AMD) is the leading cause of irreversible visual loss in the elderly [1]. It is attributable to degenerative tissue alterations occurring at the interface between the neural retina and underlying choroid [2,3]. AMD can be divided into atrophic and exudative forms. The atrophic form is more common and accounts for approximately 80% of AMD cases. However, the exudative form accounts for the majority of advanced cases [4]. The disease etiology is multifactorial—i.e., in addition to a substantial genetic component [5], aging, smoking, high body mass index, hypertension, and hypercholesterolemia predispose to AMD [6–11]. Choroidal neovascularization and leakage from the blood vessels are diagnostic markers for exudative AMD. Oxidative stress, ischemia, or inflammation can induce proliferative processes in the choroidal endothelial cells, which then evokes the growth of new blood vessels into subretinal or retinal space [2,12]. A thickening, calcification, and fragmentation of Bruch’s membrane may predispose to the development of choroidal neovascular membranes. The harmful new blood vessels that are diagnostic for exudative AMD grow through Bruch’s membrane, where they can disrupt the membrane and leak blood or fluid into the subretinal pigment epithelial space [3,4]. This can evoke damage to the photoreceptor layer and result in vision abnormalities or even total loss of vision [3,4].

The importance of genetic risk factors, specifically of those related to the complement system, has been highlighted in several studies. An association has been observed between the Y402H polymorphism (rs1061170) of the complement factor H gene and AMD in several populations [13–19]. Furthermore, an association between the LOC387715/HTRA1 locus and AMD in populations of different origin has been documented [20–28]. One common polymorphism (rs2230199) in the complement component 3 has also been associated with AMD [29,30]. Other putative candidates include genes related to fatty acid metabolism, such as apolipoprotein E [31,32], ATP-binding cassette, subfamily A, member 4 [33,34], and elongation of very long chain fatty acids-like 4 [18], but their roles in the pathogenesis of AMD are controversial.

Since dysregulated neovascularization is involved in the pathogenesis of AMD, the angiogenesis regulators represent interesting candidate genes. Tenomodulin (TNMD) is a type II transmembrane glycoprotein containing a C-terminal domain with homology to chondromodulin-I, which is a cartilage-derived angiogenesis inhibitor [35–37]. During the late developmental phase of mouse embryo, *TNMD* mRNA is expressed in the tendon of extraocular muscles, cornea, sensory retina, sclera, lens epithelial cells at the equator, and in the differentiating secondary lens fiber cells [38]. TNMD has been reported to inhibit angiogenesis by hindering endothelial proliferation and tube formation [38,39], but this has not been confirmed in vivo since the *TNMD*-deficient mice did not exhibit any vascular abnormalities when examined by oxygen-induced retinopathy [40]. Recent results suggest that single nucleotide polymorphisms (SNPs) of *TNMD* are associated with obesity, disturbed glucose metabolism, and conversion from impaired glucose tolerance to type 2 diabetes [41] as well as with elevated serum levels of systemic immune mediators [42] and serum lipoproteins [43]. Due to these connections with vascularization, inflammation, lipid metabolism, and obesity, *TNMD* is an interesting potential new candidate gene for AMD. In addition, the diaphanous *2* Drosophila homolog gene (*DIAPH*), located in the same cytogenetic band Xq-22, has been linked to AMD in a study by Zheng et al. [44]. In the present study, we investigated the associations of *TNMD* SNPs with AMD.

## Methods

### Subjects

The study population consisted of 475 Finnish subjects (162 men, 313 women). A total of 89 men and 175 women had exudative AMD, and 18 men and 25 women had atrophic AMD. Patients with choroidal neovascularization attributable to AMD were given their diagnosis based on fundus photographs and fluorescein angiography in the Department of Ophthalmology of Kuopio University Hospital or Helsinki University Hospital. The control group (no signs of AMD in fundus photographs) consisted of 55 men and 113 women. All participants were over 65 years old and did not have diabetes mellitus. The study was approved by the Ethics Committee of the Helsinki University Eye and Ear Hospital and the tenets of the Declaration of Helsinki were followed. All participants signed an informed consent form. Eligibility criteria was based on biomicroscopy examination, fundus photographs and fluorescein angiography which were performed before the study. The blood samples were stored in −80 °C until DNA isolation. DNA was extracted from peripheral blood leucocytes by salt precipitation.

### Genotype analysis

Six markers covering 75% of the common sequence variation with r^2^>0.8 in the coding region of *TNMD* (15 kb) and 10 kb upstream and downstream from the coding region (35 kb) were selected with the Tagger algorithm [45]. The markers rs2073163 and rs1155974, associated with an increased risk of type 2 diabetes [41] and elevated concentrations of serum acute phase reactants [42] in our previous studies, were included in the selection procedure.

Genotyping was performed with TaqMan Allelic Discrimination Assay according to the manufacturer’s instructions using ABI Prism 7000 sequence detector (Applied Biosystems, Foster City, CA). The genotyping success rate was 98.5% for rs7890586, 99.6% for rs1204384, and 100% for the markers rs11798018, rs5966709, rs2073163, and rs1155974. The error rate for genotyping was calculated by repeating a subset of randomly selected samples representing 6.3% of the study cohort. The error rate was 0% for all markers.

### Statistics

Haploview software [46] was used for linkage disequilibrium (LD) and Hardy–Weinberg equilibrium analysis, and association studies were performed with SPSS 14.0 for Windows (SPSS, Chicago, IL). The association of the *TNMD* SNPs with the prevalence of AMD was tested with logistic regression analysis. Due to the X-chromosomal location, a genderwise analysis was performed. In women, the association with the prevalence of AMD was assessed with additive, dominant (major allele homozygotes versus other genotypes), and recessive (minor allele homozygotes versus other genotypes) models. The genderwise differences in minor allele frequencies (MAF) were assessed with the χ^2^-test. Haplotype analyses were performed with Thesias 3.1 software [47]. Correction for multiple hypothesis testing was performed with false discovery rate using Q-value 1.0 software. π_0_ was estimated with the bootstrap method using λ range from 0 to 0.9 by 0.05 [48]. The false discovery rate for each p-value <0.1 was reported as *q*.

## Results

**Figure 1:**
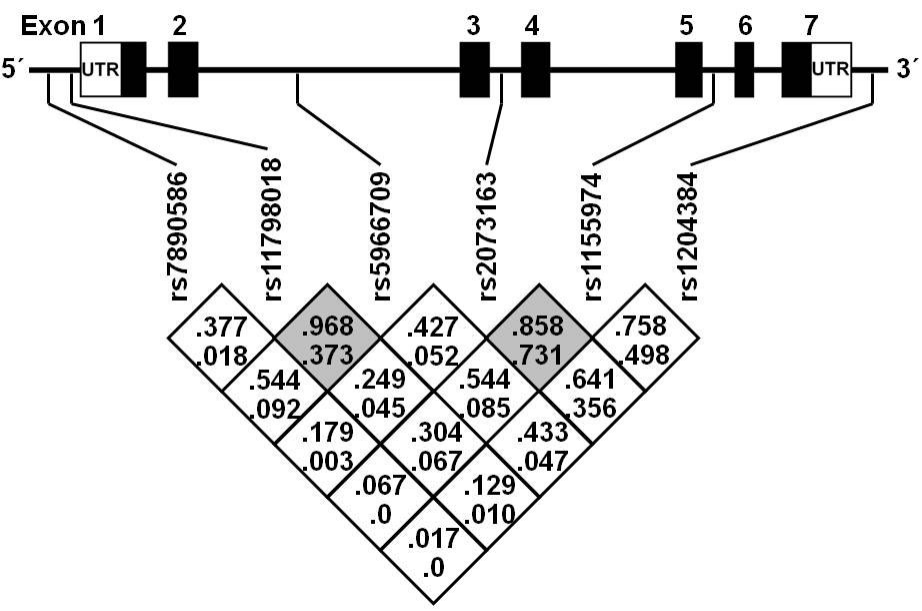
Location of the selected markers along the *TNMD* gene and their pairwise linkage disequilibrium pattern, indicated by D'- (upper) and r2-values (lower). Two haploblocks, defined by solid spine of linkage disequilibrium are denoted by gray shading. The first haploblock consists of markers rs11798018 and rs5966709, and the second consists of rs2073163 and rs1155974.

All markers, except rs7890586 (p=0.002) were in Hardy–Weinberg equilibrium. This exception was unlikely due to a genotyping error, as confirmed by genotyping a random subsample of the study population. The pairwise LD pattern and positions of the markers are presented in Figure 1. The genderwise minor allele frequencies are shown in Table 1.

**Table 1:** The genderwise minor allele frequencies of the genotyped markers.

**Marker (minor allele)**	**Minor allele frequencies** **(Men)**	**Minor allele frequencies (Women)**	***p***
rs7890586 (A)	0.175	0.055	0.00007
rs11798018 (A)	0.395	0.386	0.869
rs5966709 (T)	0.383	0.197	0.0001
rs2073163 (C)	0.315	0.279	0.462
rs1155974 (T)	0.346	0.241	0.036
rs1204384 (T)	0.304	0.198	0.024

The minor allele frequencies (MAF) of rs11798018 and rs2073163 did not differ between the genders. A minor difference was observed in the MAF of rs1204384 and rs1155974 while the MAF of rs7890586 and rs5966709 differed more significantly between men and women. Markers rs7890586, rs2073163, and rs1155974 were associated with AMD in women.

In women, the markers rs7890586 and rs1155974 were associated with total prevalence (atrophic or exudative form) of AMD, and a trend was observed with rs2073163. The odds ratios (OR) of the heterozygous women were similar to those observed among women who were major allele homozygotes (data not shown). In contrast, the women homozygous for the minor alleles had significantly different ORs for having AMD than women with other genotypes. In comparison to women with other genotypes, the women who were homozygous for either of the minor alleles rs1155974-TT or rs2073163-CC, had 2.6 fold or 1.9 fold risk for having AMD, respectively (Appendix 1). In addition, women with the rare rs7890586-AA genotype displayed a significantly smaller risk for having AMD than the women with the other genotypes (odds ratio=0.083, Appendix 1).

In more specific terms, these differences were due to the unequal prevalence of exudative AMD in the genotype groups. The women with rs2073163-CC genotype had a 2.1 fold risk (p=0.038) for exudative AMD, and the women with rs1155974-TT genotype had a 2.6 fold risk (p=0.022) in comparison to the women with other genotypes of the same markers (p values refer to the recessive model; Appendix 1). The women with rs7890586-AA genotype had a significantly smaller risk for AMD than women with rs7890586-TT or rs7890586-TA genotypes (p=0.002; recessive model, Appendix 1). The OR of heterozygous women did not differ between those observed among the women who were homozygous for the major allele (Appendix 1). None of the markers were associated with prevalence of AMD among men (Table 2). The false discovery rate was less than 10% for all of the abovementioned associations and less than 5%, apart from that of rs2073163 with total prevalence of AMD (*q*=0.067).

**Table 2:** The associations of the selected markers of the *TNMD* gene with exudative AMD in men.

**Marker**	**Genotype**	**Number of cases/controls**	**OR (95%CI)**	**p**
rs7890586	GG	78/41	1 (reference)	0.112
	AA	11/12	0.482 (0.196–1.187)	

rs11798018	CC	58/31	1 (reference)	0.292
	AA	31/24	0.690 (0.347–1.374)	

rs5966709	GG	57/31	1 (reference)	0.359
	TT	32/24	0.725 (0.362–1.441)	

rs2073163	TT	60/41	1 (reference)	0.365
	CC	29/14	1.415 (0.668–3.001)	

rs1155974	CC	60/36	1 (reference)	0.808
	TT	29/19	0.916 (0.450–1.864)	

rs1204384	AA	61/39	1 (reference)	0.84
	TT	27/16	1.079 (0.516–2.256)	

Abbreviations: odds ratio (OR); confidence interval (CI). None of the markers was associated with the prevalence of AMD in men.

Haplotype analyses were performed according to the two LD-based haploblocks, one consisting of rs11798018 and rs5966709 and the other involving rs2073163 and rs1155974. Additional analyses were performed for combinations of individual markers that were associated with AMD (rs7890586, rs2073163 and rs1155974). The results of haplotype analyses were in line with the results of single marker analyses in women, but neither of these approaches revealed a haplotype that would explain the results substantially better than the individual markers (Table 3).

**Table 3:** Associations of the *TNMD* haplotypes with total AMD (exudative and atrophic) and exudative AMD in women.

**Markers**			**Frequency**	**Total AMD**		**Exudative AMD**	
**OR (95% CI)**	**p**	**OR (95% CI)**	**p**
Haploblock 1							
rs11798018	rs5966709						
A	G		0.437				
C	T		0.329	0.821 (0.541- 1.244)	0.35	0.918 (0.618–1.365)	0.674
C	G		0.23	0.921 (0.627–1.353)	0.681	0.853 (0.557–1.306)	0.465
Haploblock 2							
rs2073163	rs1155974						
T	C		0.637				
C	T		0.287	1.411 (0.972–2.047)	0.319	1.431 (0.979–2.091)	0.064
Markers associated with AMD in women							
rs7890586	rs2073163	rs1155974					
G	T	C	0.513				
G	C	T	0.288	1.318 (0.875–1.986)	0.187	1.330 (0.877–2.017)	0.18
A	T	C	0.084	0.487 (0.241–0.986)	0.046	0.475 (0.226–0.999)	0.05

Haploblocks 1 and 2 are defined on the basis of solid spine of LD. In addition, a 3-marker haplotype was constructed of those markers that were associated with AMD in women. The haplotype analyses did not reveal a haplotype that would explain the results substantially better than the single-marker analyses. Abbreviations: odds ratio (OR); confidence interval (CI).

## Discussion

According to the results of the present study, genetic variation in the *TNMD* gene is associated with the prevalence of AMD in women. The genotypes rs2073163-CC and rs1155974-TT that associated with a higher prevalence of exudative AMD among women in the present study (Appendix 1), were linked with higher serum concentrations of macrophage migration inhibitory factor and chemokine, CC motif ligand 5 in women [42]. The same markers were associated with higher serum acute phase reactant concentrations among men [42]. In addition, the rs7890586-AA genotype was associated with a lower prevalence of exudative AMD among women (Appendix 1), but due to the low frequency of this genotype (16 out of 313 women), this finding must be interpreted cautiously. We did not observe associations with atrophic AMD, which was likely due to the small number of cases as the main aim of the study was to detect genetic risk factors for exudative AMD. In addition to single-marker analyses, haplotype analyses was performed, but they did not provide additional information (Appendix 1 and Table 3). This may be because the distribution of haplotypes that contained the individual risk alleles was almost identical to the individual allele frequencies. Since there were some missing genotypes, the number of people in the haplotype analysis was slightly lower than in the single marker analyses. This may also account for the weaker associations observed in the haplotype analyses.

The gender difference may arise from the genetic locus (Xq-22). The expression and function of X-chromosomal genes differ between genders, partially because of the variation in gene dosages and the random inactivation of one of the X-chromosomes in women [49]. Since the expression levels of *TNMD* mRNA in the adipose tissue of women are double that encountered in men [50], it seems that the *TNMD* locus is able to escape the X-inactivation. The *TNMD* locus is also relatively distant from the X-inactivation center. Genomic imprinting can also result in different gene expression levels, since women are normally mosaic for maternal and paternal active X-chromosomes; men harbor only the maternal X-chromosome and therefore express the maternally inherited genes in that locus [51]. In addition, the cellular microenvironment can differ between men and women, due to differences in hormone levels and gene expression [52]. However, we cannot rule out the possibility that the study was underpowered to detect these associations in men. The observed differences in proportions were smaller than 0.1, and the number of men included in this study population was probably not high enough to detect differences of this magnitude. The MAF distribution of rs2073163 was similar in both genders and therefore the association of rs2073163 with AMD does not result from the imbalance in the allele distribution between men and women. Gender differences were observed in the MAF of rs1155974 and rs7890586. It is difficult to speculate whether the associations of rs1155974 and rs7890586, which were observed in women, result from gender differences in MAFs; differences of equal magnitude were observed with two other markers (rs5966709 and rs1204384) that were not associated with the prevalence of AMD.

In vivo experiments in mice with oxygen-induced retinopathy have revealed that a lack of *TNMD* does not result in abnormal angiogenesis or retinal neovascularization [40]. Since the regulation of angiogenesis is a multifactorial process, it is possible that other factors might have compensated for the absence of *TNMD.* The age-associated factors can also vary between species. In addition, mice lack the normal macula and cones and therefore the regulation of retinal neovascularization in that species might be different from that in the human eye. In exudative AMD, neovascularization is evoked by a proliferation of abnormal choroidal blood vessels behind the retina. The multifactorial etiology of vascular dysregulation in AMD is not fully understood, but the expression levels of many stimulatory and inhibitory regulators of neovascularization are known to be altered during pathogenesis [12,53,54]. For example, vascular endothelial growth factor (VEGF), is known to be involved in choroidal neovascularization [54] and accordingly, VEGF-blocking compounds are emerging as highly successful treatments for exudative AMD [55–58]. This provides further evidence for the importance of angiogenesis regulators in AMD and supports the theory of a disrupted balance between stimulators and inhibitors of neovascularization in the pathogenesis of exudative AMD.

The genetic association studies on the role of VEGF polymorphisms in exudative AMD have delivered conflicting results [59–62], but interestingly, polymorphisms of the gene encoding antiangiogenic pigment epithelial growth factor have been linked to AMD [63,64]. In relation to these previous association studies with angiogenesis regulators, the associations between *TNMD* sequence variation and exudative AMD point to an interesting hypothesis on the regulatory role of TNMD in choroidal neovascularization and exudative AMD. It is important to note that the false discovery rate for the associations observed in women was low, suggesting that *TNMD* is a promising new candidate gene for AMD. The same cytogenetic band has been linked to AMD by Zheng et al., who also reported gender-specific associations with the *DIAPH2* [44]. Therefore, despite the lack of a replication sample, our results are supported by the findings of this previous study. We recognize the need for the replication of these findings in other study populations. Functional studies are needed to reveal the potential mechanisms accounting for these associations.

## Appendix 1. The association of *TNMD* SNPs with AMD in women.

The associations of the selected markers of the *TNMD* gene with the prevalence of total (athropic or exudative) and exudative AMD are reported according to additive and recessive (minor allele homozygotes versus other genotypes) models. False discovery rate (q) is indicated only for p<0.1. The markers rs7890586, rs2073163, and rs155974 were associated with total and exudative AMDTo access the data, click or select the words “Appendix 1.” This will initiate the download of a pdf that contains the file. False discovery rate *q* is reported only for p<0.1. Abbreviations: odds ratio (OR); confidence interval (CI).
